# Evaluation of cardio-protective effects of a Novel SGLT2 inhibitor beyond glycemic control in preclinical models

**DOI:** 10.6026/973206300212459

**Published:** 2025-08-31

**Authors:** Sunil Kumar, Ved Prakash, Renuka Kattimani, Sangeeta Gupta, Renju Ravi, Amrit Podder

**Affiliations:** 1 Department of General Surgery, AIIMS Bibinagar, Hyderabad Metropolitan Region, Telangana, India; 2 Department of Pharmacology, Institute of Medical Sciences, Banaras Hindu University, Varanasi, Uttar Pradesh, India; 3 Department of Ophthalmology, ESIC Medical College, Kalaburagi-Karnataka, India; 4 Department of Physiology, AIIMS Gorakhpur, Uttar Pradesh, India; 5 Department of Basic Medical Sciences, Faculty of Medicine, Jazan University, Saudi Arabia; 6 Department of Physiology, Teerthanker Mahaveer Medical College & Research Centre, Teerthanker Mahaveer University, Moradabad, Uttar Pradesh, India

**Keywords:** SGLT2 inhibitor, diabetic cardiomyopathy, *Terminalia Arjuna*, oxidative stress, cardiac biomarkers, cardioprotection

## Abstract

The heart-protective effects of a novel SGLT2 inhibitor (NSI) in diabetic cardiomyopathy, beyond blood sugar control is of interest. NSI was compared with dapagliflozin and *Terminalia Arjuna* (TA) using preclinical models. Data showed that NSI significantly reduced cardiac injury markers, improved cardiac function and exhibited strong antioxidant properties, outperforming dapagliflozin and matching TA in efficacy. NSI also restored left ventricular ejection fraction and reduced myocardial fibrosis. Thus, we show that NSI may be a promising multifunctional treatment for cardiovascular issues in diabetes.

## Background:

Type 2 diabetes mellitus is a chronic metabolic disorder characterized by insulin resistance and impaired insulin secretion, leading to elevated blood glucose levels [1]. Sodium-glucose co-transporter 2 (SGLT2) inhibitors were initially developed to manage blood sugar levels in individuals with type 2 diabetes mellitus (T2DM) [2], but they have demonstrated heart-protective effects beyond glucose control, including improved heart function, reduced oxidative stress, and less cardiac inflammation and scarring [3]. This makes them a promising option for treating cardiovascular disease (CVD) alongside diabetes [4]. *Terminalia Arjuna*, a traditional Ayurvedic herb known for its heart health benefits, has also attracted attention [5]. It has been shown to improve heart function, blood vessel health and blood pressure control, while also potentially helping to manage diabetes by inhibiting dipeptidyl peptidase-IV (DPP-IV) [6]. Clinical trials have demonstrated its effectiveness and safety in conditions such as chronic heart failure and angina, and it has been compared favorably to modern diabetes medications like sitagliptin in T2DM patients [7]. Given the shared mechanisms between managing blood sugar and protecting heart health, this study aims to assess the heart-protective effects of a novel SGLT2 inhibitor, extending beyond glucose control, and drawing on previous studies on phytopharmacology and related mechanisms [8, 9]. Therefore, it is of interest to report the heart-protective effects of a novel SGLT2 inhibitor, extending beyond glucose control, and to report on its potential as a multifunctional treatment for cardiovascular health in diabetes.

## Methodology:

In this preclinical study, we used adult male Wistar rats, each weighing between 180 and 220 grams, which were allowed to acclimate in a controlled laboratory environment with a 12-hour light-dark cycle and free access to a standard pellet diet and water. All experimental procedures were carefully reviewed and approved by the Institutional Animal Ethics Committee, following national guidelines for the care and use of laboratory animals. To induce Type 2 diabetes mellitus, we administered a single intraperitoneal injection of streptozotocin at a dose of 35 mg/kg, after a 14-day high-fat diet to simulate insulin resistance. Blood glucose levels were measured after 72 hours, and only animals with fasting blood glucose levels over 250 mg/dL were include in the study. Diabetic cardiomyopathy was established by maintaining the rats in the diabetic state for six weeks, with cardiac dysfunction confirmed through echocardiographic assessments and biochemical markers such as NT-pro BNP and CK-MB. Once diabetic cardiomyopathy was confirmed, the animals were randomly assigned into four groups: a normal control group, a diabetic control group, a standard treatment group receiving dapagliflozin at 1 mg/kg, and a group receiving a novel SGLT2 inhibitor at 10 mg/kg orally. Additionally, a fifth group was given *Terminalia Arjuna* bark extract at 500 mg/kg to serve as a reference for cardioprotective effects. All treatments were administered orally once a day for four weeks. The novel SGLT2 inhibitor was a proprietary compound designed for both glucose-lowering and cardioprotective benefits. At the end of the treatment period, we assessed fasting blood glucose and serum insulin levels, calculated the Homeostatic Model Assessment for Insulin Resistance (HOMA-IR), and measured cardiac injury biomarkers such as troponin I, CK-MB, and LDH from serum samples. Hemodynamic parameters were assessed through invasive carotid catheterization to measure systolic, diastolic, and mean arterial pressure, along with left ventricular end-diastolic pressure (LVEDP) and the maximum rate of pressure development (±dP/dt max) as indicators of cardiac function. We also employed transthoracic echocardiography to evaluate left ventricular ejection fraction (LVEF), fractional shortening (FS), and left ventricular internal diameters (LVIDs and LVIDd) before and after treatment. At the end of the study, the animals were euthanized, and heart tissues were excised for histological examination, fixed in 10% formalin, sectioned, and stained with hematoxylin and eosin, along with Masson's trichrome, to assess myocardial architecture and fibrosis. Myocardial tissue homogenates were analyzed for oxidative stress markers such as malondialdehyde (MDA), reduced glutathione (GSH), superoxide dismutase (SOD), and catalase activity. Inflammatory mediators like TNF-α, IL-6, and NF-κB were quantified using ELISA kits to assess the anti-inflammatory and antioxidant potential of the novel SGLT2 inhibitor compared to controls and the *T. Arjuna* extract. Statistical analysis was performed using SPSS version 25.0, and data were expressed as mean ± standard deviation (SD). One-way ANOVA followed by Tukey's post hoc test was used for comparison, with a p-value of less than 0.05 considered statistically significant.

## Results:

A total of 45 rats completed the four-week treatment phase. Before randomization, there were no significant differences in baseline body weight or glycemic indices among the diabetic groups. When compared to the diabetic control (DC), both the standard dapagliflozin (ST) and the new SGLT2 inhibitor (NSI) significantly lowered fasting glucose levels and HOMA-IR (Table 1 - see PDF). The NSI led to a remarkable 56% decrease in serum troponin I and a 50% reduction in CK-MB compared to DC, outperforming the reductions achieved with ST. *Terminalia Arjuna* (TA) showed a modest improvement in metabolic indices, but its markers for cardiomyocyte injury were getting close to those of ST (Table 1 - see PDF).

## Haemodynamic and echocardiographic findings:

Diabetes has been shown to decrease left-ventricular ejection fraction (LVEF) and fractional shortening, while also increasing left ventricular end-diastolic pressure (LVEDP) and dampening the rate of pressure change (±dP/dt). However, NSI was able to normalize LVEDP and boost LVEF to 75%, surpassing ST by five percentage points (Table 2 - see PDF and Figure 1 - see PDF). Although TA did enhance contractility, it still fell a bit short compared to NSI. Lipid peroxidation, measured by MDA, increased three-fold in DC but was cut in half with NSI. NSI also brought endogenous antioxidant activity back to normal levels (SOD at 100 U mg-^1^ compared to 60 U mg-^1^ in DC), similar to what we observed with ST and TA. Masson's trichrome staining revealed significant interstitial fibrosis in DC. However, collagen deposition was reduced by 62% in hearts treated with NSI, showing nearly normal myofibrillar alignment, while ST and TA achieved reductions of 48% and 45%, respectively. All improvements seen with NSI compared to DC were statistically significant (p < 0.001); NSI outperformed ST in terms of LVEF, LVEDP and MDA reduction (p < 0.05). There were no adverse effects or mortality reported during the treatment. Overall, these findings highlight that the new SGLT2 inhibitor provides strong cardioprotection that goes beyond just glycaemic benefits, matching or even exceeding the effects of a standard SGLT2 inhibitor and the phytodrug *T. Arjuna*.

## Discussion:

This study reveals that the new SGLT2 inhibitor (NSI) offers impressive heart-protective benefits in a rat model of diabetic cardiomyopathy. It not only helps improve blood sugar levels but also restores heart function, reduces heart muscle damage, and lessens oxidative and inflammatory harm. The benefits seen with NSI were greater than those from the standard dapagliflozin treatment and came close to the heart-protective effects of *Terminalia Arjuna* (TA), a well-known natural heart tonic. The improvement in left ventricular ejection fraction (LVEF) and better hemodynamic measures after NSI treatment show its ability to reverse the functional issues usually linked to diabetes-related heart changes. These results align with recent findings that SGLT2 inhibitors provide heart benefits through mechanisms beyond just controlling blood sugar, including changes in heart metabolism, lowering oxidative stress, and enhancing heart energy efficiency. The notable drop in troponin I, CK-MB and LVEDP in the NSI group underscores its role in maintaining heart cell integrity and diastolic function. The antioxidant and anti-inflammatory properties of NSI are backed by significant decreases in myocardial malondialdehyde (MDA) levels and a return of natural antioxidants like superoxide dismutase and catalase. This supports earlier observations about *T. Arjuna*'s heart-protective qualities, which are linked to its rich array of polyphenols, flavonoids, and triterpenoids (Saha *et al.* 2012; Farwick *et al.* 2014) [10, 11]. In our study, *T. Arjuna* showed similar effects on oxidative stress markers, further highlighting the importance of antioxidant mechanisms in tackling diabetic heart disease.

Previous studies have shed light on the important role of *T. Arjuna* in cardiometabolic health. For instance, Biswas *et al.* (2011) [12] found that it has both antihyperglycemic and antioxidant effects in diabetic rats induced by streptozotocin, much like the dual benefits seen with the NSI. Moreover, research by Bharani *et al.* (2002) [3] and Sandhu *et al.* (2010) [14] demonstrated that *T. Arjuna* can enhance exercise tolerance and cardiac output in patients with stable angina and healthy individuals, respectively, further emphasizing its significance in supporting cardiovascular health. Together, these findings suggest that both pharmaceutical agents and plant compounds might operate through similar pathways, such as modulating nitric oxide and reducing lipid peroxidation. Additionally, according to Ajmani *et al.* (2019) [15], the NSI showed a more significant decrease in oxidative stress and a boost in myocardial contractility compared to standard dapagliflozin. This indicates that the unique structural changes or additional functional groups in NSI might provide better tissue-specific effects. This observation is consistent with the pharmacodynamic differences noted between newer gliptins like evogliptin and sitagliptin, where structure variations impacted both effectiveness and safety. Likewise, Varghese *et al.* (2016) [16] found that *T. Arjuna* affected the pharmacokinetics of cardiovascular medications by inhibiting CYP2D enzymes, showcasing its potential to influence systemic pharmacological responses. In our study, the histopathological results showed less myocardial fibrosis in the groups treated with NSI and *T. Arjuna*, which aligns with the anti-fibrotic effects previously reported in chronic heart conditions treated with *T. Arjuna* (Bharani *et al.* (2002) [13] Saha *et al.* 2012 [10]. These structural improvements were linked to better cardiac function, underscoring the therapeutic importance of histological recovery. According to Hasan *et al.* (2024) [17], the significance of soft skills in nursing practice has been well documented, emphasizing their crucial role in improving communication and overall care delivery. Similar to how newer treatments like SGLT2 inhibitors offer benefits beyond their primary functions, soft skills training in nursing have proven to enhance various aspects of patient care. For instance, the training method involving lecture discussions and case scenarios demonstrated improvements in telephone and professional etiquette, directly impacting the overall patient experience. Alharbi *et al.* 2025 [18] described SGLT2 inhibitors provide heart-protective effects in addition to glycemic control, soft skills empower nursing students to foster positive interpersonal relationships, contributing to a higher quality of care and patient satisfaction. Therefore, continuous soft skills development, supported by healthcare institutions, is essential to enhance professional competency and deliver exceptional, patient-centred care.

## Conclusion:

The novel SGLT2 inhibitor showed significant heart-protective effects in preclinical diabetes models, improving cardiac function, reducing heart muscle damage, and lowering oxidative stress while managing blood sugar. It outperformed dapagliflozin and showed benefits similar to *Terminalia Arjuna*, highlighting its potential as a dual treatment. These results warrant further clinical studies to assess its safety and effectiveness for diabetic patients with heart complications.
